# Changes in Difficulties in Emotion Regulation Following Eating Disorders Treatment: Relevant Prospective Implications for Treatment Outcome

**DOI:** 10.3390/nu17213470

**Published:** 2025-11-03

**Authors:** Tânia F. Rodrigues, Lucero Munguía, Roser Granero, Isabel Sánchez, Jessica Sánchez-González, Jessica Jimenez-de Toro, Laura Galvez, Cristina Artero, Susana Jiménez-Murcia, Paulo P. P. Machado, Fernando Fernández-Aranda

**Affiliations:** 1 Psychotherapy and Psychopathology Research Labortary, Psychology Research Centre (CIPsi), School of Psychology, University of Minho, 4704-553 Braga, Portugal; b7784@psi.uminho.pt (T.F.R.);; 2 Clinical Psychology Department, University Hospital of Bellvitge, 08907 Barcelona, Spain; lmunguia@idibell.cat (L.M.);; 3 CIBER Fisiopatología Obesidad y Nutrición (CIBERObn), Instituto de Salud Carlos III (ISCIII), 08907 Barcelona, Spain; roser.granero@uab.cat; 4 Psychoneurobiology of Eating and Addictive Behaviours Group, Neurosciences Programme, Bellvitge Biomedical Research Institute (IDIBELL), 08908 Barcelona, Spain; 5 Department of Psychobiology and Methodology, Autonomous University of Barcelona, 08193 Barcelona, Spain; 6 Department of Clinical Sciences, School of Medicine and Health Sciences, University of Barcelona, 08907 Barcelona, Spain

**Keywords:** eating psychopathology, difficulties in emotion regulation, treatment outcome, longitudinal, CBT

## Abstract

**Background**: Difficulties in emotion regulation (ER) are commonly described in eating disorders (ED), but few studies report its impact on ED treatment outcome. The main goal of this study was to investigate the patterns of change in difficulties in ER among ED-diagnosed female patients who received a Cognitive Behavioral Therapy (CBT) treatment. **Methods**: Participants (*N* = 74; Mage = 29.2; *SD* = 11.5) completed pre- and post-treatment questionnaires to assess difficulties in ER, disordered eating symptoms, general psychopathology, and personality traits. **Results:** Considering ED treatment outcome status, 24.3% of patients displayed a poor outcome, while 28.4% achieved partial remission, and 47.3% achieved full remission. Most of the patients who achieved full remission (80%) reported decreases in difficulties in ER, while only 55.6% of the participants displaying poor outcomes reported improvements in ER. The results from the structural equation modeling (SEM) suggest that the risk of poor outcome was directly related to increased post-treatment difficulties in ER, while improvements in the severity index of global psychopathology was related to increased pre-treatment difficulties in ER. **Conclusions:** Better ED treatment outcomes are associated with higher levels of ER improvements. Future prospective studies are warranted to establish which ER components may positively influence the recovery of ED-diagnosed patients.

## 1. Introduction

Eating disorders are prevalent mental health conditions with serious medical implications, including the risk of mortality [1], characterized by pervasive disturbances in eating and/or eating-related behaviors [2].

Difficulties in ER are central to understanding the etiology and maintenance across eating psychopathology [3,4,5,6,7]. In particular, ER refers to the employment of processes through which individuals regulate which, when, and how emotions are experienced [8]. The multidimensional model of ER developed by Gratz and Roemer [9], considers four main dimensions of ER: (a) the awareness and understanding of one’s emotions; (b) the acceptance of one’s emotions; (c) the selection of goal-driven adaptive ER strategies; and (d) the ability to inhibit impulsive behaviors while experiencing emotional distress.

The functional link between difficulties in ER and disordered eating behaviors and attitudes is widely recognized (see Racine and Horvath [10] for a thorough description). Overall, ED-diagnosed individuals seem to present greater difficulties in ER in comparison to healthy controls [3,11,12]. In a network meta-analysis using data from 19,734 participants, the results consistently indicated positive associations between eating psychopathology and the use of maladaptive ER strategies [4]. According to Brockmeyer et al. [12], patients diagnosed with anorexia nervosa restrictive type (AN-R), anorexia nervosa binge-eating/purging type (AN-BP), and bulimia nervosa (BN) display comparable overall difficulties in ER, only inferior to patients diagnosed with binge eating disorder (BED). Wolz and collaborators [3] found significant differences across ED subtypes concerning overall difficulties in ER, but no differences were found concerning specific dimensions of ER (emotional awareness and clarity and the ability to refrain from impulsive behaviors under emotional distress).

The evidence reinforcing the relationship between difficulties in ER and eating psychopathology prompted the development of prospective studies to investigate the putative role of ER as a predictor of ED treatment outcome. In studies conducted with ED-diagnosed patients [5,6,7,13,14,15,16,17], decreases in emotion dysregulation after psychological treatment were concomitant to ED symptom decrease. These results held, regardless of the treatment modality or ED type (CBT, enhanced CBT, integrative cognitive–affective therapy, or dialectical–behavioral therapy were delivered to AN-, BN-, or BED-diagnosed participants) [18]. Accordingly, in another prospective study, patients with lower baseline difficulties in ER exhibited higher rates of post-treatment remission, and patients with poorer treatment outcome showed heightened baseline emotion dysregulation [19]. In another prospective study, Mallorquí-Bagué et al. [20] observed significant improvements in difficulties in ER following CBT treatment for ED-diagnosed patients. In addition, patients with good and poor treatment outcomes differed in terms of the non-acceptance of emotional states and limited access to adaptive ER strategies. Racine and Wildes [21] conducted a prospective study with AN-diagnosed female patients (who received intensive inpatient and outpatient care), and their results indicated that lower and higher levels of difficulties in emotion regulation predicted decreased or increased post-treatment eating psychopathology, respectively. On the other hand, eating psychopathology did not predict pre–post changes in ER; therefore, the authors suggested that difficulties in ER played a maintenance role in eating psychopathology but not the opposite [21]. These findings seem to underscore the importance of addressing emotion dysregulation in the treatment of EDs.

According to the Academy for Eating Disorders Psychological Care Guidelines Task Force [22], treatments based on cognitive behavioral therapy (CBT), adapted according to ED subtype, are recommended as first-line psychological approaches to treatment of adults with EDs. Although some patients achieve full symptom remission, partial and non-remission are not unusual [23]. The predictors of relapse in EDs are multifactorial [24,25]. According to Miskovic-Wheatley and collaborators [26], while an early response to treatment (i.e., meaningful reduction in ED symptoms within the first half of the treatment) was linked to higher long-term remission rates, difficulties in ER were one of the factors associated with poorer therapeutic outcomes. Despite the aforementioned, limited studies explored the relationship between changes in difficulties in ER and the treatment outcome status of ED-diagnosed patients, which may prevent the development of personalized evidence-based care, according to patients’ clinical profiles [27].

Thus, the main purpose in this study was a) to characterize a sample of ED-diagnosed female patients who received CBT treatment in terms of the treatment outcome status and to b) compare participants pertaining to each treatment outcome status (poor outcome; partial outcome; and full remission) in terms of pre- to post-treatment changes in difficulties in ER. As a secondary goal, this study aimed to explore, through a path analysis, the putative contribution of baseline and post-treatment scores in difficulties in ER and ED symptomatology to treatment outcome status.

We hypothesize that (a) those participants who display poorer treatment outcome status (e.g., partial outcome and poor outcome) will present significantly lower changes in scores in difficulties in ER, contrary to participants who achieve full remission status; we also anticipate that (b) higher baseline and post-treatment difficulties in ER will contribute significantly to higher post-treatment eating and general psychopathology levels.

## 2. Methods

### 2.1. Participants

This study included a clinical ED sample recruited at a specialized ED Unit from Barcelona, Spain. Data were curated for the purpose of the current study. Given the low percentage of male participants in the sample, they were not considered for the purpose of the current study. The exclusion criterion was being under 18 years old, and the inclusion criterion was meeting the DSM-5 criteria for an ED. All participants were assessed by specialized clinical psychologists’ through a semi-structured face-to-face interview and diagnosed according to the DSM-5 criteria [28].

### 2.2. Measures

The Difficulties in Emotion Regulation Scale (DERS) [3,9] is a 36-item self-report instrument used to assess levels of difficulties in emotion regulation, across six subscales: strategies, non-acceptance, awareness, impulse, goals, and clarity. Items are rated on a 5-point Likert scale. Values of the Cronbach’s alpha varied between 0.84 and 0.93 for the DERS subscales, with a value of 0.95 for the total score.

The Eating Disorder Inventory-2 (EDI-2) [29,30] is a 91-item self-report instrument used to assess ED-related symptoms, across 11 subscales: drive for thinness, body dissatisfaction, bulimia, ineffectiveness, perfectionism, interpersonal distrust, interoceptive awareness, maturity fears, asceticism, impulse regulation, and social insecurity. Items are rated on a 6-point Likert scale. Values of the Cronbach’s alpha varied between 0.63 and 0.87 for the EDI-2 subscales, with a value of 0.95 for the total score.

The Symptom Checklist-90-Revised (SCL-90-R) [31,32] is a 90-item self-report instrument used to assess psychological distress, across nine symptom dimensions: somatization, obsessive–compulsive, interpersonal sensitivity, depression, anxiety, hostility, phobic anxiety, paranoid ideation, and psychoticism. It derives three global indices: Global Severity Index (GSI), Positive Symptom Total (PST), and Positive Symptom Distress Index (PSDI). Items are rated on a 5-point Likert scale. Values of the Cronbach’s alpha varied between 0.84 and 0.91 for the SCL-90-R subscales, with a value of 0.98 for the total score.

The Temperament and Character Inventory-Revised (TCI-R) [33,34] is a 240-item self-report instrument used to assess temperaments (harm avoidance, novelty-seeking, reward dependence, and persistence), and character dimensions (self-directedness, cooperativeness, and self-transcendence), referred to herein as personality traits. Items are rated on a 5-point Likert scale. Values of the Cronbach’s alpha varied between 0.80 and 0.92 for the TCI-R subscales.

### 2.3. Ethics

This study was conducted in agreement with the institution’s Ethics Committee board and with the Declaration of Helsinki. Participants signed an informed consent form.

### 2.4. Treatment and Treatment Outcome

All patients received CBT-based treatment. BN- and BED-diagnosed patients received 16 weekly standardized outpatient group CBT-based sessions, in which eating monitoring, normalization of eating patterns, training in problem-solving strategies, cognitive restructuring, ER, self-esteem, body image improvement, and relapse prevention were addressed (treatments are described in detail in Fernández-Aranda et al. [35]). In the case of AN, treatment was focused on achieving nutritional restoration (outpatient individual treatment and outpatient day hospital treatment).

After treatment completion, all patients were classified in terms of treatment outcome status by specialized clinical psychologists: full-remission, partial remission, and poor outcome. Full remission was considered in the complete absence of ED symptoms for at least the last four consecutive weeks; partial remission reflected substantial but not complete symptom improvement (e.g., cessation of compensatory behavior but persistence of fear of gaining weight); and poor outcome comprised patients that met the full criteria for an ED diagnosis by the end of treatment or those who abandoned treatment (see Fernández-Aranda et al. [35,36].

### 2.5. Data Analysis

Stata18 for Windows was used to perform the statistical analyses of the data [37]. Preliminary analyses (e.g., assessment of the data response distribution) were conducted. Descriptive statistics were performed to characterize the sample and the variables in the study. Cronbach’s alphas were calculated to assess the internal consistency of the variables. Comparisons across ED diagnostic groups were conducted through the chi-square test (χ^2^) for categorical variables and analysis of variance (ANOVA) for continuous variables. Pre–post change scores were calculated. The comparison of the pre–post change scores concerning the clinical profile of participants in each treatment outcome group was based on the analysis of covariance (ANCOVA), adjusted for age and ED subtype (the diagnostic type was included as a binary control variable, coded 0–1, for the patients diagnosed with AN versus patients diagnosed with BN or BED). The effect size for the ANCOVAs was estimated using the standardized Cohen’s-*d* coefficient, deeming mild–moderate effect sizes as |*d*| > 0.50, and large–high effect sizes as |*d*| > 0.80, at the expense of simply interpreting the *p*-value (considering the sample size). The post hoc observed power was calculated, and Finner’s method [38] was used as a correction test to account for the multiple significance test procedures and avoid consequent increases in Type-I errors. With regard to the assumptions underlying the ANCOVA, it should be noted that this is a robust statistical method. Empirical and simulation studies have demonstrated that it generally upholds appropriate Type I error rates even under moderate violations of its assumptions, including unequal group sizes, deviations from normality, and heteroscedasticity [39]. This robustness supports the use of ANCOVA to examine pre–post differences across treatment outcome groups, while controlling for relevant covariates, even in studies with relatively small or unbalanced samples.

A path analysis model was tested, assessing the relationship between the pre- (Time 1) and post-treatment (Time 2) scores in difficulties in ER (DERS) and eating severity (EDI-2) and their contribution to poor treatment outcome (poor outcome) and to global psychological distress (SCL-90-R—GSI). This procedure was employed through structural equation modeling (SEM), assuming that all parameters were freely-estimated (no initial fixed value). The maximum-likelihood (ML) method was used to estimate the model, controlling for the effects of age and ED subtype. To achieve the most parsimonious model with increased statistical power, an initial complete model including all the direct and indirect effects was fitted. After that, the model was re-specified and re-adjusted without non-significant parameters. The goodness-of-fit indices considered were non-significant chi-square (χ^2^) test; the root mean square error of approximation (RMSEA), with values deemed adequate when <0.08; Bentler’s Comparative Fit Index (CFI > 0.90); Tucker–Lewis Index (TLI > 0.90); and the standardized root mean square residual (SRMR < 0.10) [40]. Although the sample size is relatively small (*N* = 74), the use of path analysis in this work is justified for several reasons. First, the tested model was theoretically driven and parsimonious, including a limited number of observed variables and paths, which reduces the risk of model overfitting. Second, recent methodological studies have shown that path analysis can yield stable and unbiased estimates with small samples when the model is simple. Third, our main goal was to explore the plausibility of the hypothesized relationships, rather than to perform a complex structural model or to generalize the results beyond our sample. Finally, while SEMs have been largely used in behavioral science research, considerations about the sample size requirements for these models seems to rely on outdated rules-of-thumb, and current Monte-Carlo studies have observed that the sample requirements fit into a very broad range (from 30 to 460, depending on the analysis characteristics) and that solutions that meet fitting at a given sample size seem to remain stable when tested with larger sample sizes [41].

## 3. Results

### 3.1. Descriptive Statistics and Baseline Mean Score Differences Across ED Subtypes

The sample comprised *N* = 74 participants (*M*_age_ = 29.2, *SD* = 11.5), who fulfilled the criteria for AN-R (*n* = 34), AN-BP (*n* = 14), BN (*n* = 14), and BED (*n* = 12). The majority of participants were single (*n* = 58, 78.4%), had completed secondary education (*n* = 32, 43.2%), were currently studying or employed (*n* = 48, 64.9%), and pertained to mean-low to low socioeconomic position indexes (*n* = 58, 78.3%). The sociodemographic and clinical data are displayed in Table 1.

The descriptive statistics for the sociodemographic variables (upper section) are presented in Table 1. No differences across ED groups were observed, except for age (BED-diagnosed patients were older in comparison to AN-diagnosed patients). Baseline means, standard deviations, and differences across ED subtype are displayed at the bottom of Table 1. The AN-R group was characterized by the lowest mean scores in the EDI-2 subscales drive for thinness, body dissatisfaction, and bulimia and the highest mean scores in the TCI-R persistence and self-directedness domains. The AN-BP group registered the highest mean scores for the DERS lack of emotional clarity dimension and the lowest mean scores in the TCI-R reward dependence subscale. BN-diagnosed patients presented the lowest mean scores regarding the DERS lack of emotional clarity dimension and the highest mean scores in both the EDI-2 drive for thinness and TCI-R reward dependence subscales. Finally, BED-diagnosed patients displayed the highest mean scores concerning the EDI-2 body dissatisfaction and bulimia subscales and the lowest mean scores in the TCI-R persistence and self-directedness subscales.

### 3.2. Treatment Outcome Distribution for the Total Sample and Across ED Subtypes

Considering the total sample, *n* = 35 patients (47.3%) achieved full remission, *n* = 21 (28.4%) achieved partial remission, and *n* = 18 patients (24.3%) displayed poor treatment outcome. Statistically significant differences were found across ED subtype (*p* = 0.015). The best therapeutic results (full-remission) were obtained among BED-diagnosed patients (*n* = 10; 83.3%), followed by BN-diagnosed (*n* = 10; 71.4%), AN-R-diagnosed (*n* = 11; 32.4%), and AN-BP-diagnosed (*n* = 4; 28.6%). Poor treatment outcome was higher among AN-diagnosed patients, followed by the BN group (*n* = 2; 14.3%) and the BED group (*n* = 0; 0.0%) (Table 2).

### 3.3. Pre–Post Changes in Difficulties in Emotion Regulation Scales Across Treatment Outcome Groups

**Figure 1:**
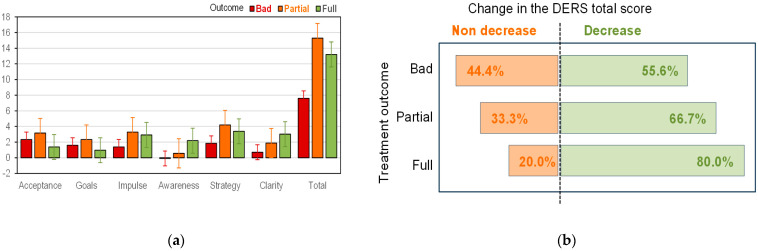
Pre to post change scores in difficulties in ER (DERS scores). Note: (**a**) mean change in the DERS scores comparing the pre to post assessment; (**b**) percentage of patients who achieved a decrease and non-decrease in the DERS scores. Results are stratified by treatment outcome and adjusted to age and ED subtype (*N* = 74).

Table 3 displays pre–post change scores in difficulties in emotion regulation (DERS), across treatment outcome status—full remission, partial remission, and poor outcome—adjusted to age and ED subtype (for a visual representation see Figure 1). The examination of the *p*-values indicated the absence of statistically significant differences across groups. The inspection of Cohen’s *d* coefficients revealed mild to moderate effect sizes between the full remission and the poor outcome groups, concerning lack of emotional awareness and clarity (subscales of the DERS).

The first graph in Figure 1a shows the mean change (decrease) in the score of each DERS scale, stratified according to the overall outcome of the intervention (the bar chart represents the adjusted mean scores from Table 3). Decreases in the DERS scores indicate improvements in ER skills or decreased difficulties in emotion regulation. For example, for the total score of the questionnaire, patients with a poor outcome in the intervention achieved a mean decrease of 7.6 points, those in partial remission achieved a mean decrease of 15.3 points, and patients in full remission achieved a mean decrease of 13.2 points.

The second graph in Figure 1b displays the percentage of patients in the sample who presented pre to post decreases/increases in the DERS scores, according to the treatment outcome group. Again, decreases in DERS scores represent improvements in ER, whereas increases indicate a higher degree of difficulties in ER. Participants pertaining to the full remission group presented the highest percentage of decrease (80.0%), followed by participants in the partial remission group (66.7%) and participants in the poor outcome group (55.6%). Conversely, 44.4% of the patients in the poor outcome group did not improve in terms of difficulties in ER.

### 3.4. Pre–Post Changes in Eating Psychopathology, Overall Psychopathology, and Personality Traits Across Treatment Outcome Groups

Table 4 displays the pre–post change scores in eating psychopathology (EDI-2), overall psychopathology (SCL-90-R), and personality traits (TCI-R) across treatment outcome status—full remission, partial remission, and poor outcome—adjusted to age and ED subtype.

Statistically significant differences with moderate to large effect sizes were found between the poor outcome and the full remission groups, concerning the EDI-2 total score, and the body dissatisfaction, interoceptive awareness, interpersonal distrust, ineffectiveness, and social insecurity subscales. Significant differences with moderate to large effect sizes were found between the poor outcome and the partial remission groups, concerning the EDI-2 total score and the body dissatisfaction subscale. Significant differences with moderate to large effect sizes were found between the partial remission and the full remission groups, concerning the interpersonal distrust subscale of the EDI-2.

Concerning overall psychopathology (SCL-90-R), the examination of the *p*-values indicated the absence of statistically significant differences across groups. Yet, the inspection of Cohen’s *d* coefficients revealed mild to moderate effect sizes concerning the subscales assessing interpersonal sensitivity and paranoid ideation, the PSDI index (poor outcome versus full remission), and phobic anxiety (poor outcome versus partial remission and poor outcome versus full remission).

Concerning personality traits (TCI-R), statistically significant differences with moderate to large effect sizes were found between the poor outcome and the full remission groups, regarding the subscales of harm-avoidance, reward dependence, and self-directedness.

### 3.5. The Association Between Pre–Post Changes in Difficulties in Emotion Regulation and Post-Treatment Clinical Profile

The effect sizes for the associations between pre–post changes in overall difficulties in ER (DERS) and the post-treatment scores in the EDI-2, SCL-90R, and TCI-R (partially controlled for age and ED subtype) are displayed in Table 5. The inspection of the results indicates that improvements in difficulties in ER (higher pre–post change scores) are associated with improvements in the clinical profile of the participants, towards a better functionality (lower post-treatment levels of eating psychopathology severity, overall psychopathology, and personality traits).

### 3.6. Path Analysis

**Figure 2:**
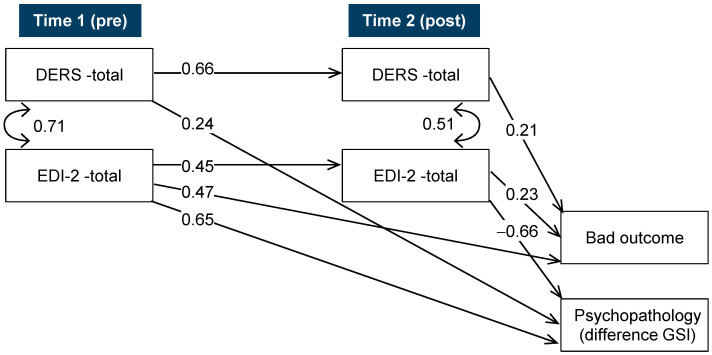
Path diagram with standardized coefficients. Note: results obtained are adjusted to age and ED subtype; only significant coefficients are retained in the final model (*N* = 74).

In the SEM displayed in Figure 2, the effects between pre- and post-treatment scores in difficulties in ER (DERS) and eating psychopathology (EDI-2) were explored. In addition, their prospective contribution to poor treatment outcomes (poor outcome) and global psychological distress (SCL-90-R—GSI) was tested. A final re-specified model with a good adjustment to the data (χ^2^ = 7.98, *p* = 0.631, RMSEA = 0.002, CFI = 0.999, TLI = 0.987, SRMR = 0.036) was achieved. No significant cross-sectional effects were found between the DERS and the EDI-2 scores. The analysis of the standardized coefficients indicated that the risk of a poor outcome was directly related to increased post-treatment DERS scores and increased EDI-2 pre- and post-treatment scores. Furthermore, higher pre–post change in GSI scores, i.e., largest improvement in the severity index of global psychopathology, was related to lower post-treatment EDI-2 scores and higher pre-treatment scores in the EDI-2 and in the DERS.

## 4. Discussion

The first goal in this study was to characterize a sample of ED-diagnosed female patients who received CBT treatment in terms of treatment outcome status and to compare participants pertaining to each treatment outcome status (poor outcome; partial outcome; and full remission) in terms of the pre- to post-treatment changes in difficulties in ER.

Previously, Sloan and collaborators [18] gathered results, consistently indicating that ED patients who received psychological treatment displayed post-treatment improvements in ER, concomitant with improvements in ED symptomatology. In the present study, pre–post changes in dimensional and overall ER difficulties were compared across treatment outcome groups. The results showed mild to large effect sizes between two particular groups of interest: poor outcome and full remission. Concerning the awareness and clarity dimensions of the DERS, participants in the full remission group displayed higher pre–post change scores. Despite the absence of significant pre–post changes in the DERS across treatment outcome groups (which may be partly explained by the sample size), those patients with partial or full ED symptom remission trended towards larger magnitudes of change with regard to their overall ability to regulate emotional states. These results are in line with previous prospective studies in which patients presented dual symptomatic improvements (for reviews, see Racine and Horvath [10] and Sloan et al. [18]). Interestingly, patients in the partial remission group evidenced higher magnitudes of change in dimensions of the DERS, in comparison to the full remission group (except in the awareness and clarity dimensions). This may be partially explained by lower baseline scores in the partial remission group, generating thus higher magnitudes of change after treatment. Conversely, the lowest changes in scores in the acceptance and goals dimensions of the DERS were observed within the full remission group. One explanatory hypothesis may be that participants who achieved better therapeutic results displayed higher baseline acceptance and goals scores, which may indicate the protective role of these ER dimensions to treatment efficacy. In terms of clinical implications, the results may suggest the value of early screening of patients with lower baseline acceptance and goals ER dimensions, along with a therapeutic focus on promoting acceptance towards emotional states and clarification of patients’ values-based goals.

Pre- to post-treatment change scores concerning other variables under study were compared across treatment outcome status. As expected, statistically significant differences were found with regard to the EDI-2 subscales. Worthy of note, the pre–post change scores in the interoceptive awareness dimension increased linearly, as patients moved from the poor outcome to the full remission group. In addition, post-treatment interoceptive awareness scores were negatively associated with the magnitude of change in the impulse dimension of the DERS, suggesting that a diminished tendency to act rashly upon negative emotions may relate to an increased ability to identify interoceptive cues post treatment. These results seem to be aligned with previous studies that highlighted the central role of interoceptive awareness as an “external field” maintenance symptom in ED [42].

Worth mentioning, mild-large effect sizes were found between the poor outcome and the full remission groups, with regard to the Positive Symptom Distress Index (PSDI). Patients with poorer treatment outcomes displayed slight pre–post changes in this dimension of psychological functioning, while patients in remission displayed higher pre–post changes. The PSDI offers insights into the overall distress per symptom reported by a patient, i.e., more than a measure of symptom frequency, it reports on the extent to which these symptoms cause particular distress. These results may suggest that patients with better treatment outcomes seem to report less perceived interference of symptoms as assessed by the SCL-90-R, which was not true with regard to the GSI dimension, which assesses the intensity of psychological symptoms and distress, but in which no significant differences were found across treatment outcome groups. These results may be clinically relevant, partly because they seem to suggest that patients may experience lower levels of distress and functional impairment, regardless of symptomatic reduction, which may be associated with the enhancement of emotion regulation skills, which theoretically augments an individual’s self-perceived ability to cope in the presence of challenging and negative-laden situations/emotions.

The same pattern was verified with regard to the TCI-R dimensions. In particular, the poor outcome and full remission groups differed with mild to large effect sizes concerning the TCI-R character and temperamental dimensions of harm avoidance, reward dependence, and self-directedness, with higher pre–post changes observed as patients transitioned from poor to good treatment outcome groups. Of note, patients belonging to the poor outcome group not only did not improve but also displayed an inverse tendency towards symptom aggravation. Additionally, post-treatment TCI-R dimensions were associated with pre–post changes in specific dimensions of the DERS, with mild to large effect sizes. These results may suggest that individual phenotypes relate to specific dimensions of difficulties in ER and are consistent with previous studies describing a cluster of ED patients with particular symptomatic presentations, characterized by higher emotional dysregulation, overall psychopathology, and specific personality traits [19,43,44,45]. Once again, routine assessment of patients may be clinically meaningful to patients’ recovery, inasmuch as early screening of lower magnitudes of change in the TCI-R dimensions may inform a targeted therapeutic focus.

Comparisons across ED categories were established for the baseline scores. Worthy of mention and in line with previous studies [12,46,47], difficulties in ER were transversal across ED types, with one exception with regard to the clarity dimension of the DERS, with significantly lower scores in the BN group.

Another objective in this study was to explore, through a path analysis, the contribution of baseline and post-treatment scores in difficulties in ER and ED symptomatology, to treatment outcome status.

In the path analysis, no significant cross-sectional effects were found between the DERS and the EDI-2 scores. These results seem to suggest that improvements in the DERS scores are not simply caused by therapeutic improvements in eating psychopathology and vice-versa, and independent processes may operate with regard to trajectories of change in ED symptomatology and difficulties in ER [18]. Notwithstanding, the standardized coefficient paths indicated that the risk of poor outcome was associated with higher post-treatment DERS scores. In addition, higher pre–post change GSI scores (improvements in global psychopathology) were directly related to higher baseline DERS scores. Even though the results are not conclusive in determining predictive variables for ED treatment outcome, they contribute to affirm the contributive role of high levels of difficulties in ER to poor ED treatment outcomes. Further, these results partially support previous research [43] establishing a linear and positive relationship between difficulties in ER and ED symptom severity.

Overall, the results demonstrated that patients who do not improve their ER skills are more likely to display poorer treatment outcomes. In addition, the treatment outcome groups showed heterogeneity concerning eating-related symptomatology, difficulties in ER, overall psychopathology, and personality traits.

In terms of clinical implications, promising approaches to the treatment of difficulties in ER include contextual-based therapeutic approaches (e.g., acceptance- and compassion-based interventions), adjunctive treatments (e.g., extended psychoeducational modules; mental health mobile applications), neurocognitive training (e.g., biofeedback-powered tools, serious games), among others [48]. In addition, tailored and cost-effective therapeutic protocols may enhance ED therapeutic gains, as opposed invariably applying a “measure that fits them all” [49].

The results of our study may have different clinical implications; considering individual differences regarding ER may help to define ED treatment and address patients’ immediate therapeutic needs in a timely manner.

### Limitations and Future Studies

Limitations in this study must be acknowledged. First, our sample comprises only adult women; therefore, these results are not generalizable to men and adolescents. Although the wide age range and the transdiagnostic nature of the sample poses a strength due to its inclusiveness, the modest clinical sample size prevents a more in-depth analysis of the results across age group and ED-subtype and the establishment of causality without sample size and power constraints (most observed power values for the ANCOVA analyses, as shown in Table 3 and Table 4, were low). In addition, our sample was limited to AN, BN, and BED categories of EDs. Despite growing empirical evidence supporting a transdiagnostic approach to EDs, these results are not generalizable to patients diagnosed with not otherwise specified or unspecified EDs. Studies on larger clinical samples would allow testing the models’ invariance across ED diagnoses, age, and gender, as well as running path analysis with latent variables, preventing potential attenuation or inflation of the path coefficients’ estimates.

We sought to characterize the clinical profile of patients who received CBT-based treatment, across three treatment outcome groups. However, future studies using active and healthy control groups could be more informative regarding longitudinal trajectories of change. Although patients received a CBT-based treatment, its duration, delivery mode, and intensity across ED subtype differed; even though this reflects the idiosyncratic needs across ED diagnostic categories, the present results must be cautiously considered. Nevertheless, the statistical analyses in the study included ED subtype as a confound variable, to account for the putative effect of therapeutic-based variations in the results.

On another note, concerns about the factorial structure of the DERS, particularly regarding the awareness scale were previously mentioned. Although beyond the scope of this study, future studies are warranted to explore the trajectories of change in difficulties in emotion regulation, considering both the six- and the five-factor solution proposed for the DERS scale in previous studies [50,51].

## 5. Conclusions

The results in this study add to a growing body of literature suggesting that larger improvements in ER following ED treatment may be associated with more favorable ED outcomes. Patients who showed limited progress in ER tended to experience poorer therapeutic results. In addition, results from a path analysis suggest that improvements in ER and ED symptoms may evolve through partially independent mechanisms. However, higher post-treatment ER difficulties were linked to poorer outcomes, underscoring the key role of ER.

Overall, these findings seem to suggest the importance of targeting ER to enhance ED treatment efficacy. Future studies are still necessary to further establish these results and to assess the potential of adding personalized adjunctive therapeutic components to the already existent evidence-based treatments for EDs, in order to foster ER skills and enhance ED treatment outcomes.

## Figures and Tables

**Table 1:** Descriptives for the variables at baseline and comparison between ED subtypes.

	Total *N* = 74		AN-R *n* = 34		AN-BP *n* = 14		BN *n* = 14		BED *n* = 12		
	*n*	*%*	*n*	*%*	*n*	*%*	*n*	*%*	*n*	*%*	*p*
Marital status											0.055
Single	58	78.4%	30	88.2%	11	78.6%	9	64.3%	8	66.7%	
Married	10	13.5%	4	11.8%	0	0.0%	4	28.6%	2	16.7%	
Divorced	6	8.1%	0	0.0%	3	21.4%	1	7.1%	2	16.7%	
Educational level											0.638
Primary	18	24.3%	7	20.6%	5	35.7%	2	14.3%	4	33.3%	
Secondary	32	43.2%	16	47.1%	6	42.9%	5	35.7%	5	41.7%	
University	24	32.4%	11	32.4%	3	21.4%	7	50.0%	3	25.0%	
Occupational status											0.242
Unemployed	26	35.1%	13	38.2%	7	50.0%	2	14.3%	4	33.3%	
Student/employed	48	64.9%	21	61.8%	7	50.0%	12	85.7%	8	66.7%	
Socioeconomic status											0.415
Middle-high	7	9.5%	4	11.8%	0	0.0%	1	7.1%	2	16.7%	
Middle	9	12.2%	4	11.8%	3	21.4%	2	14.3%	0	0.0%	
Middle-low	22	29.7%	7	20.6%	4	28.6%	7	50.0%	4	33.3%	
Low	36	48.6%	19	55.9%	7	50.0%	4	28.6%	6	50.0%	
	*M*	*SD*	*M*	*SD*	*M*	*SD*	*M*	*SD*	*M*	*SD*	*p*
Age (yrs-old)	29.23	11.51	25.38	10.22	27.86	11.24	34.14	10.84	36.00	12.20	0.011 *
Onset of ED (yrs-old)	19.01	8.74	17.94	7.63	18.29	3.89	19.21	8.74	22.67	14.32	0.445
Duration of ED (yrs)	10.20	10.67	7.39	8.61	9.56	11.57	15.09	14.14	13.17	8.56	0.096
DERS											
Non acceptance	17.84	6.92	16.06	7.34	20.00	6.08	18.43	6.51	19.67	6.53	0.209
Goals	16.57	5.14	15.24	4.86	17.50	5.11	17.57	4.40	18.08	6.35	0.230
Impulse	15.74	6.10	14.24	5.43	16.07	6.35	17.71	5.41	17.33	7.85	0.224
Awareness	17.78	5.36	18.00	5.69	18.07	4.68	15.57	3.80	19.42	6.43	0.314
Strategy	24.32	8.30	22.82	8.51	26.36	9.35	25.07	7.40	25.33	7.61	0.532
Clarity	14.41	5.14	14.35	5.88	16.86	3.57	11.36	4.29	15.25	3.79	0.034 *
Total	106.66	26.98	100.71	30.82	114.86	23.84	105.71	21.91	115.08	21.68	0.250
EDI-2											
Drive for thinness	11.59	6.43	8.71	6.91	14.79	4.73	15.07	4.21	12.00	5.41	0.001 *
Body dissatisfaction	14.26	7.44	11.38	6.46	16.36	5.09	14.14	8.37	20.08	7.81	0.002 *
Interoceptive awareness	10.47	7.19	8.82	7.93	12.21	5.37	10.43	7.41	13.17	5.92	0.233
Bulimia	5.03	4.97	1.12	1.79	4.43	3.06	10.21	3.24	10.75	3.65	0.001 *
Interpersonal distrust	6.14	4.88	6.65	5.50	7.14	3.66	3.93	3.05	6.08	5.68	0.284
Ineffectiveness	10.54	7.35	9.68	7.55	13.21	6.91	8.14	6.96	12.67	7.04	0.186
Maturity fears	7.57	5.25	8.74	6.57	6.79	3.58	6.71	3.41	6.17	4.09	0.366
Perfectionism	4.88	4.62	4.41	3.90	4.07	5.15	6.57	5.64	5.17	4.65	0.447
Impulse regulation	4.24	4.69	3.68	4.31	4.71	5.72	4.79	4.21	4.67	5.35	0.826
Ascetic	6.01	4.07	5.29	4.03	7.00	4.47	6.14	3.94	6.75	3.93	0.522
Social insecurity	7.66	5.46	8.15	6.09	8.14	4.02	5.00	4.15	8.83	5.97	0.237
Total	88.39	40.70	76.62	45.43	98.86	31.02	91.14	32.79	106.33	38.29	0.101
SCL-90-R											
Somatization	1.47	0.93	1.28	1.01	1.74	0.98	1.47	0.79	1.69	0.77	0.361
Obsessive	1.62	0.80	1.47	0.81	1.71	0.95	1.79	0.62	1.73	0.78	0.537
Sensitivity	1.84	0.91	1.69	0.99	2.05	0.90	1.96	0.70	1.92	0.95	0.578
Depressive	2.04	0.93	1.88	1.03	2.21	0.98	2.22	0.49	2.09	1.00	0.584
Anxiety	1.43	0.85	1.21	0.83	1.66	0.92	1.67	0.75	1.50	0.88	0.221
Hostility	1.13	0.81	1.07	0.83	1.25	1.04	1.10	0.57	1.22	0.79	0.883
Phobic anxiety	0.72	0.78	0.52	0.73	0.94	0.95	0.92	0.71	0.83	0.77	0.212
Paranoia	1.27	0.84	1.16	0.78	1.44	0.88	1.24	0.94	1.39	0.89	0.713
Psychotic	1.14	0.73	1.04	0.74	1.41	0.84	1.16	0.68	1.04	0.59	0.428
GSI	1.50	0.72	1.35	0.77	1.71	0.78	1.61	0.51	1.58	0.68	0.360
PST	58.38	18.34	53.79	21.03	63.86	16.18	63.00	14.15	59.58	15.06	0.231
PSDI	2.19	0.55	2.08	0.58	2.29	0.56	2.28	0.38	2.29	0.59	0.481
TCI-R											
Novelty seeking	93.92	14.90	90.56	14.64	93.86	15.48	98.79	15.14	97.83	13.91	0.259
Harm avoidance	116.89	20.89	114.62	21.85	116.29	20.01	112.00	21.67	129.75	14.49	0.124
Reward dependence	97.27	16.93	95.71	18.10	91.71	10.42	109.36	13.15	94.08	18.53	0.022 *
Persistence	107.53	18.95	112.94	18.85	107.71	20.76	108.29	14.33	91.08	13.39	0.006 *
Self-directedness	124.97	19.76	131.79	21.31	121.14	12.90	120.93	19.90	114.83	16.40	0.036 *
Cooperativeness	136.95	13.98	136.62	15.36	135.14	14.53	139.71	9.63	136.75	14.70	0.854
Self-transcendence	63.46	15.15	60.09	14.44	65.86	18.30	68.86	13.96	63.92	13.86	0.285

*Note.* AN-R, anorexia nervosa restrictive type, AN-BP, anorexia nervosa binge-eating/purging type; BN, bulimia nervosa; BED, binge eating disorder; DERS, Difficulties in Emotion Regulation Scale; EDI-2, Eating Disorder Inventory-2; SCL-90-R, Symptom Checklist-90-Revised; TCI-R, Temperament and Character Inventory-Revised; * Bold, significant comparison.

**Table 2:** Distribution of the treatment outcome and comparison across ED subtypes.

	Total *N* = 74		AN-R *n* = 34		AN-BP *n* = 14		BN *n* = 14		BED *n* = 12		
Outcome	*n*	%	*N*	%	*n*	%	*n*	%	*n*	%	*p*
Poor outcome	18	24.3%	12	35.3%	4	28.6%	2	14.3%	0	0.0%	0.015 *
Partial remission	21	28.4%	11	32.4%	6	42.9%	2	14.3%	2	16.7%	
Full remission	35	47.3%	11	32.4%	4	28.6%	10	71.4%	10	83.3%	

*Note.* AN-R, anorexia nervosa restrictive type, AN-BP, anorexia nervosa binge-eating/purging type; BN, bulimia nervosa; BED, binge eating disorder; * Bold, significant comparison.

**Table 3:** Comparison of the pre–post changes in the DERS scores (ANCOVA adjusted to age and ED subtype).

	Descriptives (Adjusted) for the Pre–Post Changes						Pairwise Comparisons						
	Poor Outcome		Partial Remission		Full Remission		Poor Outcome		Poor Outcome		Partial Remiss.		
	*n* = 18		*n* = 21		*n* = 35		Partial Remiss.		Full Remission		Full Remission		
	*M*	*SD*	*M*	*SD*	*M*	*SD*	*p*	*|d|*	*p*	*|d|*	*p*	*|d|*	*Power*
Non acceptance	2.34	4.58	3.16	6.64	1.39	8.06	0.713	0.14	0.662	0.15	0.385	0.24	0.109
Goals	1.61	3.31	2.33	5.13	0.97	4.63	0.592	0.17	0.627	0.16	0.271	0.28	0.149
Impulse	1.39	6.00	3.28	5.71	2.92	5.34	0.288	0.32	0.380	0.27	0.825	0.06	0.151
Awareness	−0.09	3.98	0.57	4.30	2.19	4.29	0.627	0.16	0.090	**0.55 ^†^**	0.194	0.38	0.340
Strategy	1.86	6.44	4.19	6.95	3.39	7.20	0.290	0.35	0.480	0.22	0.689	0.11	0.142
Clarity	0.70	3.91	1.89	3.94	3.02	4.76	0.406	0.30	0.099	**0.53 ^†^**	0.384	0.26	0.292
Total	7.60	18.35	15.28	23.61	13.21	21.32	0.252	0.36	0.393	0.28	0.734	0.09	0.163

*Note.* Results obtained for the total sample; M: mean; SD, standard deviation; Power: observed power (computed using alpha = 0.05); ^†^ Bold, mild to large effect size.

**Table 4:** Comparison of the pre–post changes (ANCOVA adjusted to age and ED subtype).

	Descriptives (Adjusted) for the Pre–Post Changes						Pairwise Comparisons						
	Poor Outcome		Partial Remission		Full Remission		Poor Outcome		Poor Outcome		Partial Remiss.		
	*n* = 18		*n* = 21		*n* = 35		Partial Remiss.		Full Remission		Full Remission		
	*M*	*SD*	*M*	*SD*	*M*	*SD*	*p*	*|d|*	*p*	*|d|*	*p*	*|d|*	*Power*
EDI-2													
Drive for thinness	1.06	6.49	3.02	8.21	4.10	6.25	0.369	0.26	0.158	**0.50 ^†^**	0.589	0.15	0.222
Body dissatisfaction	−1.76	5.64	5.70	6.49	4.03	6.96	0.001 *	**1.23 ^†^**	0.007 *	**0.92 ^†^**	0.391	0.25	0.905
Interoceptive awareness	0.16	6.32	4.02	7.91	5.02	7.19	0.091	**0.54 ^†^**	0.031 *	**0.72 ^†^**	0.627	0.13	0.499
Bulimia	3.13	3.15	3.55	4.92	3.78	5.21	0.715	0.10	0.567	0.15	0.829	0.04	0.075
Interpersonal distrust	−0.04	3.15	1.78	4.39	4.04	3.75	0.121	0.48	0.001 *	**1.18 ^†^**	0.037 *	**0.55 ^†^**	0.899
Ineffectiveness	0.01	6.62	2.87	8.70	4.85	5.72	0.207	0.37	0.032 *	**0.78 ^†^**	0.341	0.27	0.470
Maturity fears	−1.38	4.06	0.02	5.79	0.27	4.43	0.365	0.28	0.279	0.39	0.863	0.05	0.156
Perfectionism	−0.57	2.96	0.90	3.48	1.30	3.36	0.172	0.46	0.079	**0.59 ^†^**	0.690	0.11	0.342
Impulse regulation	−0.48	3.36	0.94	5.16	0.00	3.92	0.256	0.33	0.694	0.13	0.411	0.20	0.165
Ascetic	−0.51	4.30	1.80	4.13	1.72	3.26	0.060	**0.55 ^†^**	0.064	**0.58 ^†^**	0.947	0.02	0.450
Social insecurity	−0.04	3.50	2.13	5.82	3.46	4.17	0.149	0.45	0.019 *	**0.91 ^†^**	0.330	0.26	0.547
Total	−0.07	32.13	27.20	43.75	32.94	33.57	0.019 *	**0.71 ^†^**	0.004 *	**1.00 ^†^**	0.582	0.15	0.778
SCL-90-R													
Somatization	0.23	0.76	0.39	0.89	0.47	0.87	0.567	0.19	0.375	0.30	0.743	0.09	0.112
Obsessive	0.10	0.70	0.27	0.93	0.41	0.79	0.501	0.21	0.224	0.41	0.566	0.15	0.173
Sensitivity	0.05	0.74	0.21	1.10	0.53	0.76	0.543	0.17	0.070	**0.64 ^†^**	0.196	0.34	0.371
Depressive	0.22	0.84	0.55	0.96	0.56	0.91	0.241	0.37	0.219	0.40	0.969	0.01	0.206
Anxiety	0.14	0.60	0.45	0.85	0.36	0.84	0.205	0.42	0.354	0.30	0.692	0.10	0.190
Hostility	0.19	0.74	0.18	0.59	0.21	0.68	0.945	0.02	0.928	0.03	0.863	0.05	0.252
Phobic anxiety	−0.08	0.44	0.31	0.96	0.24	0.60	0.074	**0.52 ^†^**	0.130	**0.61 ^†^**	0.735	0.08	0.370
Paranoia	−0.07	0.64	0.12	0.96	0.29	0.74	0.436	0.23	0.127	**0.52 ^†^**	0.427	0.20	0.254
Psychotic	0.14	0.57	0.45	0.80	0.26	0.61	0.130	0.44	0.553	0.20	0.305	0.27	0.260
GSI	0.13	0.56	0.37	0.80	0.40	0.62	0.246	0.35	0.176	0.47	0.852	0.05	0.226
PST	3.81	17.81	7.96	19.22	8.73	17.80	0.460	0.22	0.372	0.28	0.881	0.04	0.119
PSDI	0.13	0.39	0.35	0.66	0.39	0.57	0.195	0.42	0.128	**0.54 ^†^**	0.823	0.06	0.277
TCI-R													
Novelty seeking	−0.18	8.45	−3.87	11.71	−1.93	13.20	0.335	0.36	0.640	0.16	0.580	0.16	0.125
Harm avoidance	−3.03	19.23	4.71	19.73	11.25	16.96	0.196	0.40	0.017 *	**0.79 ^†^**	0.232	0.36	0.568
Reward dependence	1.95	13.95	−3.71	13.62	−8.00	10.18	0.144	0.41	0.010 *	**0.81 ^†^**	0.225	0.36	0.636
Persistence	−4.03	16.12	−3.36	14.80	−0.51	13.35	0.885	0.04	0.439	0.24	0.501	0.20	0.107
Self-directedness	2.05	15.71	−5.65	19.12	−8.75	18.73	0.166	0.44	0.050 *	**0.62 ^†^**	0.541	0.16	0.408
Cooperativeness	4.86	12.58	3.02	13.50	0.32	9.02	0.619	0.14	0.213	0.42	0.426	0.24	0.186
Self-transcendence	2.73	14.63	1.77	13.43	2.68	12.08	0.822	0.07	0.990	0.00	0.817	0.07	0.255

*Note.* Results obtained among the total sample; EDI-2, Eating Disorder Inventory-2; SCL-90-R, Symptom Checklist-90-Revised; TCI-R, Temperament and Character Inventory-Revised; M: mean; SD: standard deviation; Power: observed power (computed using alpha = 0.05); * Bold, significant comparisons; ^†^ Bold, mild to large effect size.

**Table 5:** Correlation matrix with pre–post changes in the DERS and clinical profile post treatment (partial coefficients adjusted to age and ED subtype).

Change in DERS⟶	Acceptance	Goals	Impulse	Awareness	Strategy	Clarity	Total
↓ Post Treatment							
EDI-2 Drive for thinness	−0.071	0.110	−0.113	0.079	−0.174	0.018	−0.104
EDI-2 Body dissatisfaction	−0.166	−0.093	**−0.240 ^†^**	0.094	**−0.259 ^†^**	0.009	−0.176
EDI-2 Interoceptive awareness	−0.152	−0.082	**−0.250 ^†^**	0.103	−0.189	−0.014	−0.172
EDI-2 Bulimia	**−0.318 ^†^**	−0.158	**−0.303 ^†^**	−0.148	**−0.260 ^†^**	**−0.238 ^†^**	**−0.382 ^†^**
EDI-2 Interpersonal distrust	−0.122	−0.229	**−0.282 ^†^**	−0.057	−0.201	−0.135	**−0.239 ^†^**
EDI-2 Ineffectiveness	−0.188	−0.173	**−0.336 ^†^**	0.053	−0.234	−0.076	**−0.250 ^†^**
EDI-2 Maturity fears	−0.092	−0.044	−0.192	0.104	**−0.258 ^†^**	0.119	−0.111
EDI-2 Perfectionism	**0.305 ^†^**	0.092	0.189	0.117	0.087	0.139	**0.235 ^†^**
EDI-2 Impulse regulation	−0.010	−0.080	−0.150	−0.047	−0.072	0.016	−0.072
EDI-2 Ascetic	−0.040	−0.105	−0.123	−0.059	−0.124	−0.082	−0.128
EDI-2 Social insecurity	−0.177	−0.234	**−0.242 ^†^**	−0.032	−0.213	−0.115	**−0.240 ^†^**
EDI-2 Total	−0.148	−0.122	**−0.278 ^†^**	0.049	**−0.261 ^†^**	−0.035	−0.217
SCL-90R Somatization	−0.106	0.170	−0.033	−0.084	−0.124	−0.024	−0.057
SCL-90R Obsessive	−0.179	−0.047	−0.201	−0.110	−0.197	−0.193	−0.215
SCL-90R Sensitivity	−0.117	−0.127	−0.213	−0.080	−0.231	−0.123	−0.211
SCL-90R Depressive	−0.177	−0.058	**−0.257 ^†^**	−0.144	**−0.292 ^†^**	−0.122	−0.234
SCL-90R Anxiety	−0.087	−0.035	−0.118	−0.127	−0.185	−0.047	−0.106
SCL-90R Hostility	−0.115	−0.028	−0.029	0.000	−0.023	−0.093	−0.038
SCL-90R Phobic anxiety	−0.041	0.126	−0.049	−0.098	−0.039	−0.060	0.004
SCL-90R Paranoia	−0.050	0.014	−0.151	0.006	−0.118	−0.020	−0.073
SCL-90R Psychotic	−0.053	−0.075	−0.185	−0.043	−0.183	−0.048	−0.133
SCL-90R GSI	−0.133	−0.009	−0.183	−0.104	−0.213	−0.094	−0.164
SCL-90R PST	−0.161	−0.071	−0.214	−0.042	**−0.264 ^†^**	−0.111	−0.181
SCL-90R PSDI	−0.161	−0.016	−0.180	−0.109	**−0.263 ^†^**	−0.116	−0.209
TCI-R Novelty seeking	0.224	−0.171	0.032	−0.102	0.020	−0.001	0.045
TCI-R Harm avoidance	−0.172	−0.115	**−0.326 ^†^**	−0.058	**−0.343 ^†^**	−0.130	**−0.313 ^†^**
TCI-R Reward dependence	0.180	**0.287 ^†^**	**0.276 ^†^**	0.015	0.193	0.158	**0.245 ^†^**
TCI-R Persistence	−0.040	0.022	0.106	−0.019	0.055	0.065	0.049
TCI-R Self-directedness	**0.268 ^†^**	**0.347 ^†^**	**0.342 ^†^**	0.069	**0.311 ^†^**	0.170	**0.333 ^†^**
TCI-R Cooperativeness	**0.248 ^†^**	**0.411 ^†^**	**0.360 ^†^**	0.019	**0.327 ^†^**	0.070	**0.322 ^†^**
TCI-R Self-transcendence	**0.245 ^†^**	**0.246 ^†^**	**0.301 ^†^**	0.048	**0.288 ^†^**	0.122	**0.306 ^†^**

*Note.* Results obtained among the total sample (*n* = 74); DERS, Difficulties in Emotion Regulation Scale; EDI-2, Eating Disorder Inventory-2; SCL-90-R, Symptom Checklist-90-Revised; TCI-R, Temperament and Character Inventory-Revised; ^†^ Bold, mild to large effect size.

## Data Availability

The data presented in this study are available on request from the corresponding author due to ethical issues, and privacy/legal matters.
